# Noninvasive evaluation of IgA nephropathy fibrosis using Doppler ultrasound

**DOI:** 10.1080/0886022X.2022.2140060

**Published:** 2022-10-28

**Authors:** Xiachuan Qin, Chaoxue Zhang

**Affiliations:** a Department of Ultrasound, The First Affiliated Hospital of Anhui Medical University, Hefei, China; b Department of Ultrasound, Nanchong Central Hospital, The Second Clinical Medical College, North Sichuan Medical College (University), Nan Chong, China

**Keywords:** IgA nephropathy, biopsy, fibrosis, nomograms, ultrasound

## Abstract

In this study, we aimed to explore the clinical value of routine color ultrasound parameters in the evaluation of tubular atrophy and interstitial fibrosis (TA/IF) in IgA nephropathy (IgAN). We enrolled 725 patients with IgAN who underwent renal biopsy at the First Affiliated Hospital of Anhui Medical University between January 2019 and May 2022. Examinations were performed to measure the routine ultrasound renal parameters and renal biopsy was done within next three days. Univariate and multivariate analyses were used to determine the correlates and the independent predictors of TA/IF. Simultaneously, a nomogram based on risk indicators was created to predict TA/IF. Univariate and multivariate analyses showed that sex (*p* < 0.001, OR = 2.538, 95%CI: 1.739–3.734), renal length (*p* < 0.001, OR = 0.927, 95%CI: 0.905–0.95), resistive index of main renal artery (*p* = 0.037, OR = 1.891, 95%CI: 1.027–3.426), peak systolic velocity of segmental renal artery (*p* = 0.58, OR = 0.975, 95%CI: 0.399–0.841), and cortex echogenicity (*p* < 0.001, OR = 3.448, 95%CI: 2.382–5.018) were independent predictors of TA/IF in IgAN nomograms, with a good *C*-index of 0.765 (95%CI = 0.727–0.803). Analyses of the calibration charts show that nomograms have good performance and clinical applicability. In our study, renal color ultrasound parameters correlated well with TA/IF in IgAN. By establishing a conventional color ultrasound prediction model, we can accurately gauge the extent of TA/IF in patients with IgAN for clinical applications.

## Introduction

IgA nephropathy (IgAN) is the most common primary glomerulonephritis in China and is the main cause of end-stage renal disease [1,2]. Reportedly, the incidence rate of IgA is 0.2–5 cases per 100,000 persons per year. Highest incidence rates are seen in East Asia while North America and Europe have lower rates [3]. Histopathology results often guide any ameliorative treatment. Many classification systems available use some criteria of biopsy scoring to predict the rate of decline in renal function, but Oxford classification is more widely used in IgAN [4,5]. Roberts used MEST criteria to predict the outcome of IgAN in 13 studies: endocapillary hypercellularity had independent prognostic value in four, segmental glomerulosclerosis in four, and tubular atrophy/interstitial fibrosis (T, referred to here as TA/IF) in 10 of the studies [6]. TA/IF is the result of chronic glomerular and tubular injury, and its degree is related to the development of ESRD [7]. It is considered a risk prediction tool in IgAN [8]. Yu et al. believe that TA/IF lesion is the most reliable indicator for predicting IgAN progression [9]. However, the diagnosis of TA/IF is based on renal biopsy, an invasive procedure unsuitable for long-term monitoring of disease progression and treatment response [10].

Therefore, a noninvasive and accurate technique is needed to detect and monitor TA/IF. Complex and practically difficult techniques of functional imaging have been tried in some studies without getting ideal results [10–14]. Clinically, routine renal ultrasound is the preferred method to evaluate and follow up IgAN morphologically, as it is simple and cost-effective. We could not find any large cohort studies evaluating the specific ultrasound parameters correlate with the indicators of IgAN TA/IF. The purpose of this study was to explore the clinical value of routine color ultrasound parameters in the evaluation of TA/IF in IgAN on a larger patient number.

## Methods

This study was approved by the institutional review committee of the First Affiliated Hospital of Anhui Medical University (PJ2022-11-29). We enrolled 725 renal biopsy-proven IgAN patients at the First Affiliated Hospital of Anhui Medical University from January 2019 to May 2022.

The inclusion criteria were as follows: (1) renal biopsy-proven IgAN; (2) at least 10 glomeruli in the specimen visible under light microscope; (3) age of the patient more than 18 years.

The exclusion criteria were: (1) acute renal damage, heart valve disease, and heart failure; (2) renal artery stenosis or urinary tract obstruction; (3) cysts or tumors in the kidneys; (4) Doppler patterns suggestive of renal artery stenosis [15].

### Ultrasound measurements

Four radiologists with a minimum of 5 years’ experience in routine ultrasound performed the examinations and acquired images (within three days before the due date of renal biopsy).

The ultrasound instruments used to acquire the images in this study included: Mindray Resona 7 (Shenzhen Mindray BioMedical Electronics Co., Ltd., Shenzhen, China); GE Vivid E9 (General Electric Co., Boston, MA); Aplia 500 (TOSHIBA, Minato, Japan); a 2–5 MHz frequency convex array transducer (C5-2) was used as a probe.

**Figure 1.**
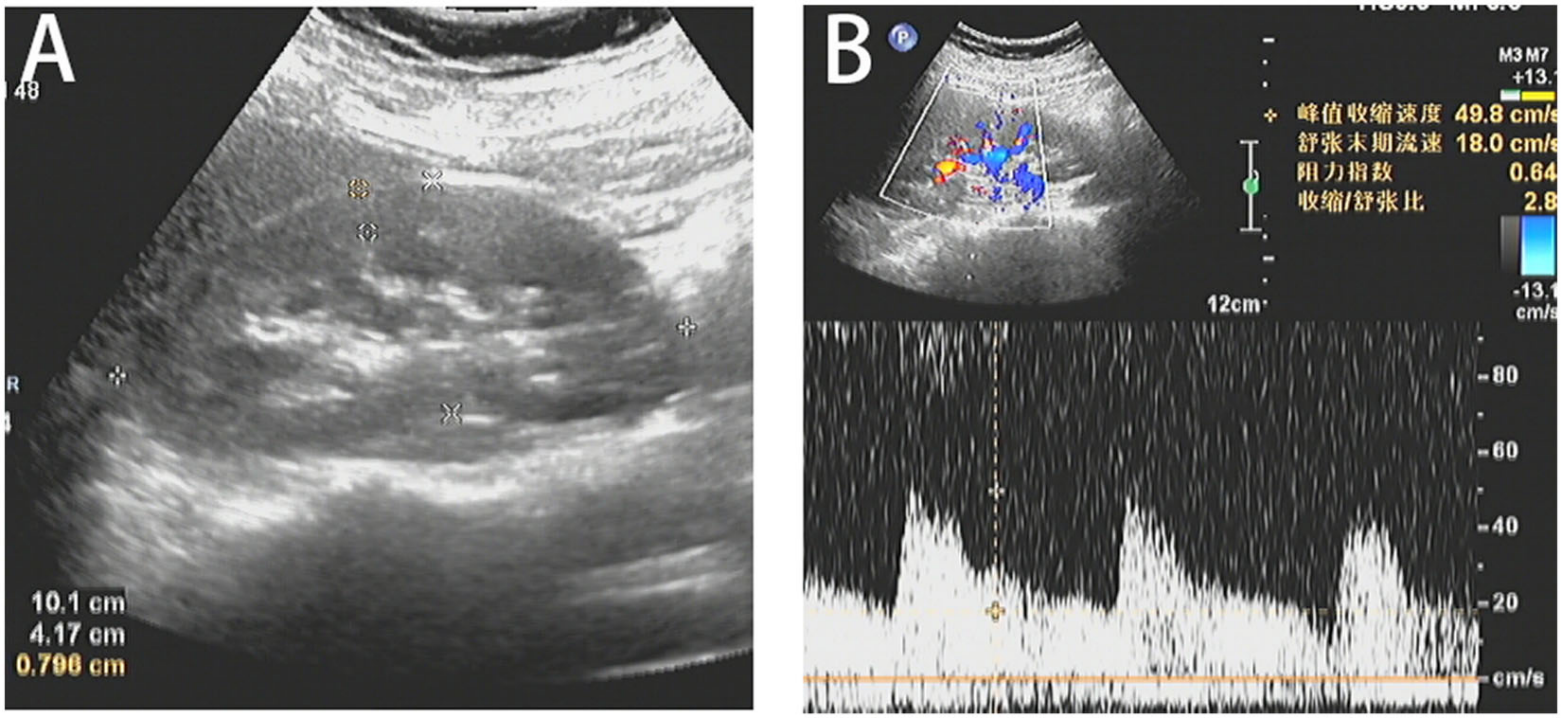
(A) The renal length, width, and cortical thickness of the right kidney measured; (B) the Doppler beam on the right MRA of PSV and RI.

The patient fasted for more than 8 h. Patient was made to lie in the supine position. The following parameters were measured by ultrasound (during end-inspiratory breath-hold): kidney size (length, width, and cortical thickness of the right kidney measured at the maximum cross section) and renal echo (echo is defined as the echo of the renal cortex equal to or stronger than that of the liver parenchyma). Images were obtained in Doppler and pulse Doppler modes. The ultrasonic probe is gently positioned in the right abdomen in the form of oblique projection, visualizing the kidney as a longitudinal image, placing the Doppler beam on the trunk of the right renal artery, measuring the peak systolic velocity (PSV) of the main renal artery (MRA), and measuring the PSV and end diastolic velocity (EDV) for an average of 2–3 times to calculate the resistance index (RI)=(PSV – EDV)/PSV (Figure 1). Intrarenal Doppler signals were obtained from two to three representative proximal segmental arteries (the first vascular branch of the MRA) of the right kidney, and RI and PSV of segmental renal artery (SRA) were calculated.

### Renal biopsy and pathological examination

The indicators of a renal biopsy are as follows: (1) acute nephritis syndrome (a, function drops sharply, acute nephritis is suspected; b, the condition does not improve after 2–3 months of treatment according to acute nephritis); (2) primary nephrotic syndrome (a, regular hormone treatment for 8 weeks has no effect; b, the puncture obtains pathology for differentiated treatment); (3) clinical diagnosis of denatured red blood cell hematuria is unclear; (4) proteinuria continues to be greater than 1 g/d, and the diagnosis is unclear.

Renal biopsy from right kidneys was performed by two experienced nephrologists within three days of renal ultrasound measurements. Light, electron, and immunofluorescence microscopies were used to examine biopsied tissues. All biopsy samples to be valid, needed to have at least 10 glomeruli each on light microscopy. MEST-C criteria put forward in Oxford classification of IgAN were used to score the following histopathologic findings: mesangial cell proliferation (M0/M1), endocapillary hypercellularity (E0/E1), segmental glomerulosclerosis (S0/S1), tubular atrophy/interstitial fibrosis (TA/IF0, TA/IF1, TA/IF2), and crescents (C) [4]. The percentage of TA/IF of cortical area were: TA/IF0: 0–25%, TA/IF1: 26–50%, TA/IF2: >50% [4].

### Statistical analysis

Due to limited number of samples, TA/IF1 and TA/IF2 cases were combined into one group. Single factor logistic regression analysis was used to analyze the correlation between ultrasonic parameters and TA/IF. The related factors (*p* < 0.1) thus discovered were analyzed by multifactor logistic regression analysis to determine the independent predictors significantly related to TA/IF. Nomograms were constructed using the variables in the final multiple logistic regression model to evaluate the indicators of TA/IF. The discriminative ability of predictive nomograms was evaluated by Harrell’s concordance index (*C*-index) [16]. *C*-index and area under curve have similar meanings. The highest value of *C*-index is 1, which means a perfect distinction and the minimum value is 0.5, representing random distinction. Calibration is used to compare the probability predicted by our nomogram with the actual probability of TA/IF. *p* < 0.05 (two-tailed) indicated significance. IBM SPSS Statistics 26 statistical software package (SPSS Inc., Chicago, IL), R language software and RMS software package (Risk Management Solutions, Inc., Newark, CA) were used.

## Results

### Clinical and pathological characteristics of patients

A total of 725 patients with IgAN (renal biopsy-proven) were included in this study with 346 male (47.7%) and 379 female (52.3%), and an average age of 39.5 ± 12.5 years. Among the cases, 541 cases were TA/IF0, while 184 were TA/IF1 and TA/IF combined. The clinical and pathological characteristics of patients are listed in Table 1.

**Table 1.** Patients’ baseline clinical characteristics.

Clinical characteristics	
Age (years)	39.5 ± 12.5
Sex (male/female)	346/379
Systolic arterial pressure (mmHg)	133.4 ± 41.6
Diastolic arterial pressure (mmHg)	86.5 ± 13.4
Hemoglobin at biopsy (g/L)	132.5 ± 18.9
Urea at biopsy (mmol/L)	6.3 ± 2.7
Uric acid at biopsy (μmol/L)	381 ± 103.9
24 h proteinuria at biopsy (g/24 h)	1.7 ± 4.3
Proteinuria at biopsy (g/L)	1.1 ± 2.3
MEST histologic score	
M1	*n* = 725 (100%)
E1	*n* = 236 (32.6%)
S1	*n* = 399 (55%)
T0	*n* = 541 (74.6%)
T1	*n* = 166 (22.9%)
T2	*n* = 18 (2.5%)
Crescents	*n* = 233 (32.1%)

M: mesangial cell proliferation; E: endocapillary hypercellularity; S: segmental glomerulosclerosis; T: tubular atrophy/interstitial fibrosis.

### Independent US factors predicting TA/IF in IgAN

Routine ultrasonic characteristics of IgAN patients are shown in Table 2. In univariate logistic analysis, age (*p* = 0.005), sex (*p* < 0.001), renal length (*p* < 0.001), PSV of MRA (*p* < 0.001), RI of MRA (*p* = 0.005), PSV of SRA (*p* < 0.001), cortex echo (*p* < 0.001), and the thickness of renal cortex (*p* = 0.076) were associated with TA/IF. The above factors with *p* < 0.1 were further included in the multivariate logistic regression. The results showed that sex (*p* < 0.001, OR = 2.538, 95%CI: 1.739–3.734), renal length (*p* < 0.001, OR = 0.927, 95%CI: 0.905–0.95), RI of MRA (*p* = 0.037, OR = 1.891, 95%CI: 1.027–3.426), PSV of SRA (*p* = 0.58, OR = 0.975, 95%CI: 0.399–0.841), and cortex echo (*p* < 0.001, OR = 3.448, 95%CI: 2.382–5.018) are independent predictors of TA/IF (Table 3). RI of MRA’ cutoff is 0.72 and PSV of SRA’ cutoff is 40 cm/s.

**Table 2.** The color ultrasonic characteristics of IgAN patients.

	T0 (*n* = 541)	T1 and T2 (*n* = 184)	*p*
Age (years)	39.1 ± 11.9	42 ± 12.9	0.005
Sex (male/female)	228/315	112/70	<0.001
Renal length (mm)	104.9 ± 8.4	100.5 ± 7.6	<0.001
Renal width (mm)	44.8 ± 6.3	44.3 ± 6.3	0.35
The thickness of RC (mm)	7.3 ± 1.4	7.0 ± 1.5	0.076
PSV of MRA (cm/s)	78.7 ± 17.7	72.9 ± 18.8	<0.001
RI of MRA	0.64 ± 0.06	0.66 ± 0.06	0.005
PSV of SRA (cm/s)	43 ± 9.5	40 ± 9.8	<0.001
RI of SRA	0.62 ± 0.06	0.62 ± 0.06	<0.001
Cortical echogenicity (increased/normal)	151/392	107/75	<0.001

RC: renal cortex; PSV: peak systolic velocity; MRA: main renal artery; RI: resistance index; SRA: segmental renal artery.

**Table 3.** The independent predictors of AT/IF on multivariate logistic regression.

	OR	95%CI	*p*
Sex	2.538	1.739–3.734	<0.001
Renal length (mm)	0.927	0.905–0.95	<0.001
RI of MRA	1.891	1.027–3.426	0.037
PSV of SRA (cm/s)	0.58	0.399–0.841	0.004
Cortical echogenicity (increased/normal)	3.448	2.382–5.018	<0.001

### Nomograph of US indicators for noninvasive diagnosis of TA/IF

**Figure 2.**
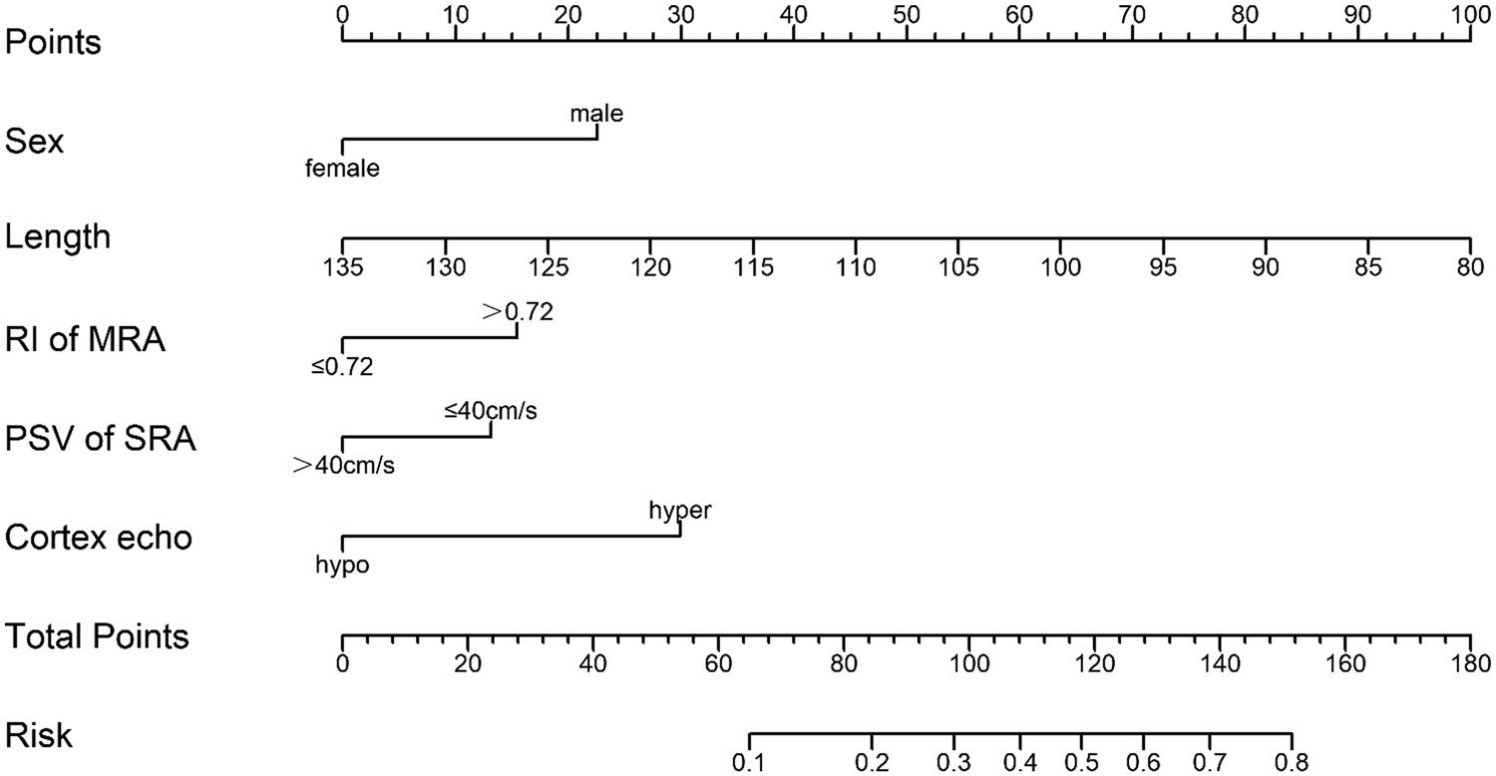
Nomogram for predicting TA/IF in IgAN based on five indicators.

**Figure 3.**
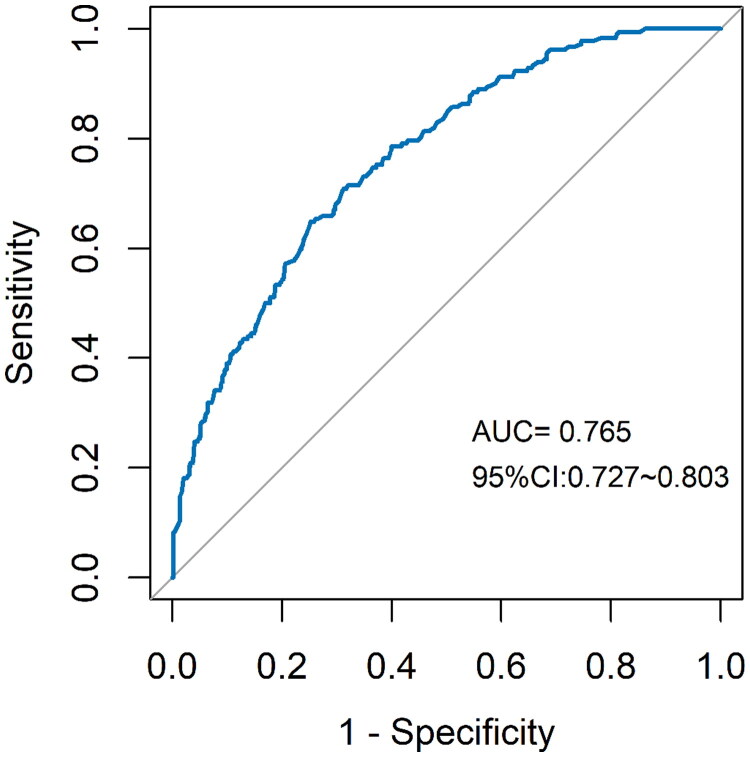
ROC curve for the predictive of ultrasonographic features. The predictive model of TA/IF in IgAN was accurate and discriminating, with AUC of 0.765.

**Figure 4.**
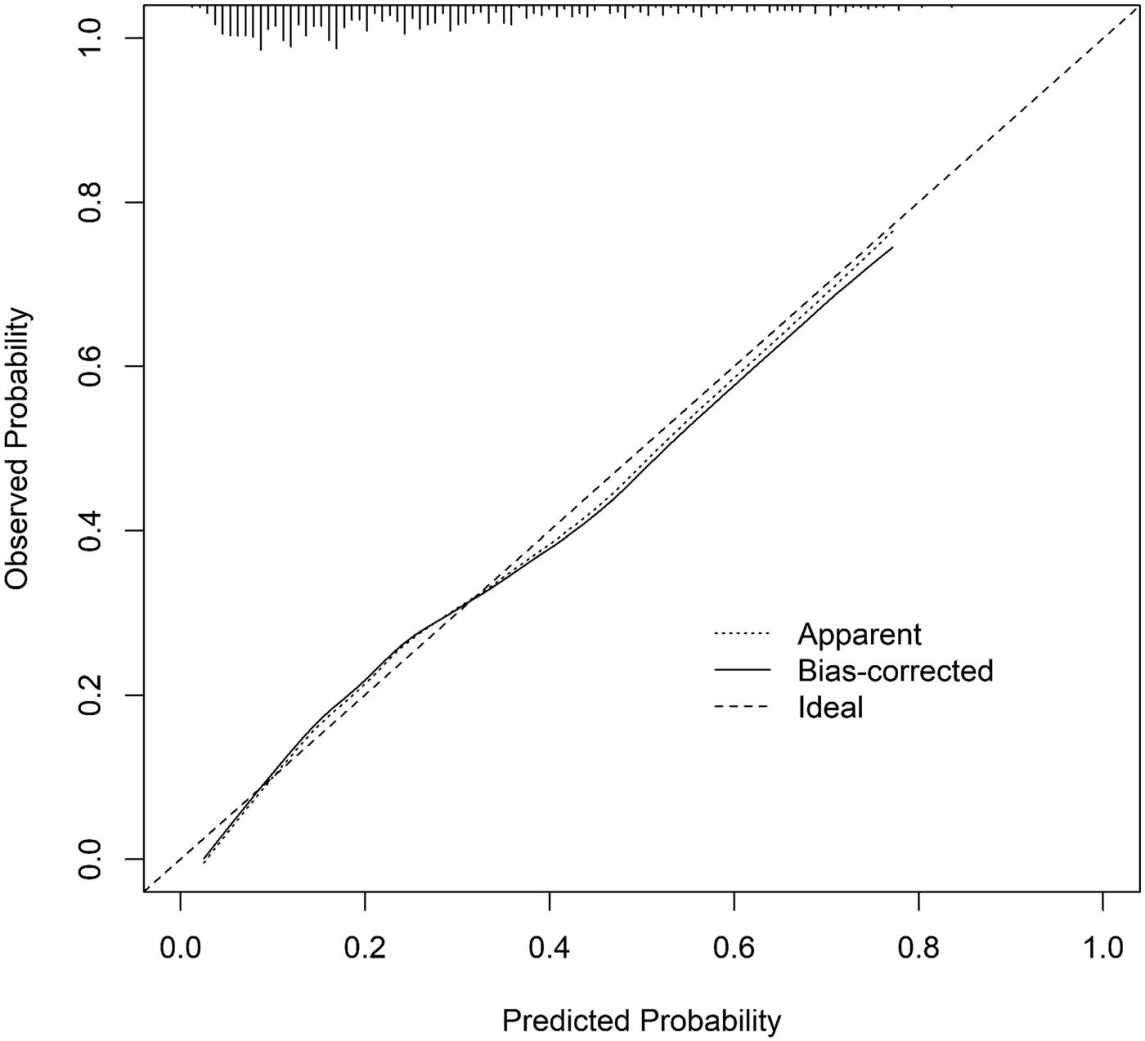
Calibration plots of nomogram for predicting TA/IF in IgAN.

Based on the results of the multivariate logistic regression model analysis of color US indicators to predict TA/IF in patients with IgAN, we established a nomogram (Figure 2). The nomogram integrates five independent variables (sex, renal length, RI of MRA, PSV of SRA, cortex echo) which are helpful for noninvasive diagnosis of TA/IF, and the degree of IgAN, where each level in the variable assigns a score according to the score level. By adding the total score and positioning it on the total score scale, the corresponding probability of TA/IF for each person can be determined. The nomogram has a good *C*-index of 0.765 (95%CI = 0.727–0.803) (Figure 3). The calibration chart also shows a good consistency between the actual probability of nomogram diagnosis TA/IF (Figure 4).

## Discussion

Based on univariate and multivariate analyses of large number of renal biopsy samples and certain ultrasound parameters in IgAN patients, this study systematically analyzed the relationship between color ultrasound parameters and TA/IF. Our results show that sex, renal length, RI of MRA, PSV of SRA, and cortex echo are the five independent noninvasive predictors of TA/IF. Moreover, we developed nomograms based on the identified indicators because nomograms can integrate all these variables and estimate the TA/IF degree of each patient separately. The nomogram model based on color ultrasound indicators showed good discrimination ability, with a *C*-index of 0.765. With this model, we can estimate TA/IF in patients with IgAN. To the best of our knowledge, this is the first large sample-size study to use color ultrasound to predict IgAN TA/IF. Irreversible damage to the nephrons, due to chronic glomerular or tubular injury, lead to TA/IF around the damaged nephrons [7]. The degree of renal fibrosis correlates with the loss of renal function and renal damage progression. In some previous studies, based on Oxford classification criteria, TA/IF was found to be the most important predictor of end-stage renal disease. Multivariate analysis revealed that the risk ratio of patients with TA/IF ≥ 1 to reach the primary endpoint is 32 compared with TA/IF0 (*p* = 0.01). Based on our experience, the Oxford classification predicts progressive kidney disease, but the degree of TA/IF is the only independent predictor [17]. Yu et al. found that S and TA/IF in the Oxford criteria of IgAN were independent risk factors affecting the progression of nephropathy [18]. Coppo et al. found that only T lesions were associated with the rate of eGFR loss in the whole cohort through a 35-year follow-up of 1130 patients with IgAN [19]. The degree of fibrosis affects the deterioration of renal function [20]. The evaluation of TA/IF is currently based on renal biopsy which has the limitations of being invasive, possible sampling errors, and provides limited spatial information. These make it unsuitable for long-term monitoring of disease progression or treatment response [7].

Male is independently associated with poor kidney outcomes [21]. Huang et al.’s research also shows that male-non-exercise population seems to be more likely to progress to the end-stage renal failure than others [22]. Wen et al. thought renal survival rates of male patients were remarkably lower than those of female patients, and male sex was identified as an independent risk factor for poor outcomes [23]. The protective anti-fibrotic and anti-apoptotic effects of estrogen in women and/or the deleterious pro-inflammatory effects of testosterone in men, coupled with unhealthier lifestyles and delayed diagnoses, might cause kidney function to decline faster in men than in women. We conducted a multivariate analysis, age was excluded, and sex was an independent predictor of AT/IF. Our study is consistent with previous studies, and men have a higher level of AT/IF.

Simplicity and convenience of color ultrasound makes it the preferred method for renal morphology and blood flow evaluation by nephrologists. Longitudinal renal length is considered to be a key indicator of chronic kidney disease because it decreases with the gradual decline of eGFR [24]. In IgAN patients, renal TA/IF gets aggravated, resulting in the reduction of renal length [25,26]. Normally, renal cortex has lower echogenicity than normal liver and spleen. In Zhang et al.’s opinion, hyperechoic is a typical feature of graft dysfunction [27]. Echo changes because of the increasing glomerulosclerosis and TA/IF. Moreover, in our study, higher echogenicity represents more severe TA/IF. RI is helpful to evaluate the general vascular condition of CKD patients and provide information about microvascular and macrovascular damage [28,29]. Li et al. thought RI had a potential role in discriminating diabetic kidney disease from non-diabetic kidney disease [30]. A high RI can predict adverse outcomes in patients with chronic kidney disease [31]. It has long been considered as a marker of the progression of renal damage [32]. Notably, RI is associated with glomerulosclerosis, tubulointerstitial changes, and arteriosclerosis [33]. With the deterioration of chronic kidney disease, renal perfusion pressure and glomerular filtration rate continue to rise, resulting in thickening of glomerular capillary wall, enhanced permeability, narrowing of vascular lumen, and increased glomerular capillary pressure. These changes eventually lead to increased forward flow resistance and other renal artery hemodynamic disorders. Consistent with previous studies, RI increased with aggravation of TA/IF. The aggravation of glomerulosclerosis and TA/IF leads to the loss of capillaries around glomeruli and renal tubules [33,34]. Simultaneously, the accumulation of pathological mesangial matrix caused by glomerulosclerosis and tubulointerstitial changes leads to the narrowing of glomerular capillaries, which may reduce the perfusion of post-glomerular capillaries, and the blood flow stagnation of peritubular capillaries in the diseased kidneys [35]. This explains, to some extent, why the flow velocity between segments was lower in T1 and T2 in our study. TA/IF being an important predictor of the progression of chronic kidney disease, its noninvasive detection in IgAN is advantageous in dynamic monitoring of disease progression so that a preventive and timely intervention can be implemented. We have established a color US prediction model which can accurately and noninvasively judge IgAN TA/IF. We believe that it has great clinical potential because it is convenient and universal.

The limitations of our study are as follows: (1) the use of time-varying covariates in proportional hazard models may produce time-varying confounding limitations in a single-race single-center design; (2) only adults are included in our analyses, so the prediction model may not be applicable to children; (3) in this study, TA/IF1 and TA/IF2 were merged, the study had limited TA/IF2 samples, which can be further studied in future research.

In conclusion, the results of our study bring out the correlation between color US parameters and TA/IF in IgAN. By establishing a US prediction model, we can accurately gauge the degree of TA/IF in patients with IgAN. This model is convenient and noninvasive and hence applicable in daily clinical practice after validation from future studies.

## References

[1] Li M, Foo JN, Wang JQ, et al. Identification of new susceptibility loci for IgA nephropathy in Han Chinese. Nat Commun. 2015;6:7270. 26028593 10.1038/ncomms8270 PMC4458882

[2] Wyatt RJ, Julian BA. IgA nephropathy. N Engl J Med. 2013;368(25):2402–2414. 23782179 10.1056/NEJMra1206793

[3] Pattrapornpisut P, Avila-Casado C, Reich HN. IgA nephropathy: core curriculum 2021. Am J Kidney Dis. 2021;78(3):429–441. 34247883 10.1053/j.ajkd.2021.01.024

[4] Markowitz G. Glomerular disease: updated Oxford classification of IgA nephropathy: a new MEST-C score. Nat Rev Nephrol. 2017;13(7):385–386. 28529339 10.1038/nrneph.2017.67

[5] Cattran DC, Coppo R, Cook HT, et al. The Oxford classification of IgA nephropathy: rationale, clinicopathological correlations, and classification. Kidney Int. 2009;76(5):534–545. 19571791 10.1038/ki.2009.243

[6] Roberts IS. Oxford classification of immunoglobulin A nephropathy: an update. Curr Opin Nephrol Hypertens. 2013;22(3):281–286. 23518465 10.1097/MNH.0b013e32835fe65c

[7] Tampe D, Schridde L, Korsten P, et al. Different patterns of kidney fibrosis are indicative of injury to distinct renal compartments. Cells. 2021;10(8):2014. 34440782 10.3390/cells10082014 PMC8392296

[8] Barbour SJ, Coppo R, Zhang H, et al. Evaluating a new international risk-prediction tool in IgA nephropathy. JAMA Intern Med. 2019;179(7):942–952. 30980653 10.1001/jamainternmed.2019.0600 PMC6583088

[9] Yu F, Zhu X, Yuan S, et al. Predictive value of sub classification of focal segmental glomerular sclerosis in Oxford classification of IgA nephropathy. Ann Med. 2021;53(1):587–595. 33825605 10.1080/07853890.2021.1897664 PMC8032344

[10] Sun Q, Baues M, Klinkhammer BM, et al. Elastin imaging enables noninvasive staging and treatment monitoring of kidney fibrosis. Sci Transl Med. 2019;11:486. 10.1126/scitranslmed.aat4865 PMC7115882 30944168

[11] Asano K, Ogata A, Tanaka K, et al. Acoustic radiation force impulse elastography of the kidneys: is shear wave velocity affected by tissue fibrosis or renal blood flow? J Ultrasound Med. 2014;33(5):793–801. 24764334 10.7863/ultra.33.5.793

[12] Wu J, Shi Z, Zhang Y, et al. Native T1 mapping in assessing kidney fibrosis for patients with chronic glomerulonephritis. Front Med. 2021;8:772326. 10.3389/fmed.2021.772326 PMC8558353 34733870

[13] Yang WQ, Mou S, Xu Y, et al. Quantitative parameters of contrast-enhanced ultrasonography for assessment of renal pathology: a preliminary study in chronic kidney disease. Clin Hemorheol Microcirc. 2018;68(1):71–82. 29036800 10.3233/CH-170303

[14] Grenier N, Poulain S, Lepreux S, et al. Quantitative elastography of renal transplants using supersonic shear imaging: a pilot study. Eur Radiol. 2012;22(10):2138–2146. 22588518 10.1007/s00330-012-2471-9

[15] Kliewer MA, Tupler RH, Carroll BA, et al. Renal artery stenosis: analysis of Doppler waveform parameters and tardus-parvus pattern. Radiology. 1993;189(3):779–787. 8234704 10.1148/radiology.189.3.8234704

[16] Harrell FE Jr., Lee KL, Mark DB. Multivariable prognostic models: issues in developing models, evaluating assumptions and adequacy, and measuring and reducing errors. Stat Med. 1996;15(4):361–387. 8668867 10.1002/(SICI)1097-0258(19960229)15:4<361::AID-SIM168>3.0.CO;2-4

[17] Yau T, Korbet SM, Schwartz MM, et al. The Oxford classification of IgA nephropathy: a retrospective analysis. Am J Nephrol. 2011;34(5):435–444. 21968096 10.1159/000332223

[18] Yu GZ, Guo L, Dong JF, et al. Persistent hematuria and kidney disease progression in IgA nephropathy: a cohort study. Am J Kidney Dis. 2020;76(1):90–99. 32197881 10.1053/j.ajkd.2019.11.008

[19] Coppo R, D'Arrigo G, Tripepi G, et al. Is there long-term value of pathology scoring in immunoglobulin a nephropathy? A validation study of the Oxford classification for IgA nephropathy (VALIGA) update. Nephrol Dial Transplant. 2020;35(6):1002–1009. 30418652 10.1093/ndt/gfy302

[20] Konieczny A, Donizy P, Gołębiowski T, et al. Clinical and histopathological factors influencing IgA nephropathy outcome. Diagnostics. 2021;11(10):1764. 34679462 10.3390/diagnostics11101764 PMC8534654

[21] Tan J, Luo X, Yang J, et al. Clinicopathological characteristics and risk factors in elderly patients with biopsy-proven IgA nephropathy. Ren Fail. 2022;44(1):1026–1036. 35766236 10.1080/0886022X.2022.2087527 PMC9246206

[22] Huang PP, Shu DH, Su Z, et al. Association between lifestyle, gender and risk for developing end-stage renal failure in IgA nephropathy: a case-control study within 10 years. Ren Fail. 2019;41(1):914–920. 31580172 10.1080/0886022X.2019.1635029 PMC6781456

[23] Wen D, Tang Y, Tan L, et al. Sex disparities in IgA nephropathy: a retrospective study in Chinese patients. Int Urol Nephrol. 2021;53(2):315–323. 32944891 10.1007/s11255-020-02631-7

[24] Petrucci I, Clementi A, Sessa C, et al. Ultrasound and color Doppler applications in chronic kidney disease. J Nephrol. 2018;31(6):863–879. 30191413 10.1007/s40620-018-0531-1

[25] van der Sande NG, Blankestijn PJ, Leiner T, et al. High ratios of kidney function to kidney size are related to mortality and kidney function decline in high-risk patients. Eur J Prev Cardiol. 2017;24(9):926–933. 28121180 10.1177/2047487317690950

[26] Jovanović D, Gasic B, Pavlovic S, et al. Correlation of kidney size with kidney function and anthropometric parameters in healthy subjects and patients with chronic kidney diseases. Ren Fail. 2013;35(6):896–900. 23751145 10.3109/0886022X.2013.794683

[27] Zhang J, Chen GD, Qiu J, et al. Color Doppler ultrasound and hemodynamics for evaluating graft dysfunction in recurrent immunoglobulin a nephropathy. Ann Transplant. 2021;26:e931736. 34413279 10.12659/AOT.931736 PMC8409140

[28] Ștefan G, Florescu C, Sabo AA, et al. Intrarenal resistive index conundrum: systemic atherosclerosis versus renal arteriolosclerosis. Ren Fail. 2019;41(1):930–936. 31599199 10.1080/0886022X.2019.1674159 PMC6807913

[29] Cianci R, Gigante A, Bagordo D, et al. Renal resistive index in IgA nephropathy and renal scleroderma vasculopathy. Microvasc Res. 2021;133:104095. 33035555 10.1016/j.mvr.2020.104095

[30] Li H, Shen Y, Yu Z, et al. Potential role of the renal arterial resistance index in the differential diagnosis of diabetic kidney disease. Front Endocrinol. 2021;12:731187. 10.3389/fendo.2021.731187 PMC8796316 35095752

[31] Parolini C, Noce A, Staffolani E, et al. Renal resistive index and long-term outcome in chronic nephropathies. Radiology. 2009;252(3):888–896. 19528356 10.1148/radiol.2523080351

[32] Fiorini F, Granata A, Noce A, et al. Index resistance in renal ultrasound: what is the clinical significance? G Ital Nefrol. 2013;30(2):1–16. 23832456

[33] Ikee R, Kobayashi S, Hemmi N, et al. Correlation between the resistive index by Doppler ultrasound and kidney function and histology. Am J Kidney Dis. 2005;46(4):603–609. 16183414 10.1053/j.ajkd.2005.06.006

[34] Kang DH, Kanellis J, Hugo C, et al. Role of the microvascular endothelium in progressive renal disease. J Am Soc Nephrol. 2002;13(3):806–816. 11856789 10.1681/ASN.V133806

[35] Matsumoto M, Tanaka T, Yamamoto T, et al. Hypoperfusion of peritubular capillaries induces chronic hypoxia before progression of tubulointerstitial injury in a progressive model of rat glomerulonephritis. J Am Soc Nephrol. 2004;15(6):1574–1581. 15153568 10.1097/01.asn.0000128047.13396.48

